# Application of expanded genetic analysis in the diagnosis of familial hypercholesterolemia in patients with very early-onset coronary artery disease

**DOI:** 10.1186/s12967-018-1737-7

**Published:** 2018-12-10

**Authors:** Ye-Xuan Cao, Na-Qiong Wu, Di Sun, Hui-Hui Liu, Jing-Lu Jin, Sha Li, Yuan-Lin Guo, Cheng-Gang Zhu, Ying Gao, Qiu-Ting Dong, Geng Liu, Qian Dong, Jian-Jun Li

**Affiliations:** Division of Dyslipidemia, State Key Laboratory of Cardiovascular Disease, Fu Wai Hospital, National Center for Cardiovascular Diseases, Chinese Academy of Medical Sciences, Peking Union Medical College, BeiLiShi Road 167, Beijing, 100037 China

**Keywords:** Familial hypercholesterolemia, Genetic testing, Very early-onset CAD

## Abstract

**Background:**

Patients with monogenic familial hypercholesterolemia (FH) have high risk for coronary artery disease (CAD). A recent FH Expert Panel suggested that FH was underdiagnosed and undertreated which needs early diagnosis. Moreover, the proportion of DNA-confirmed FH patients hospitalized with very early-onset (≤ 35 years) CAD remains uncertain.

**Methods:**

One hundred and five patients with age ≤ 35 years and LDL-C ≥ 3.4 mmol/L were tested for 9 genes (*LDLR, APOB, PCSK9, APOE, STAP1, LIPA, LDLRAP1, ABCG5/8*). Dutch Lipid Clinic Network (DLCN) and Simon Broome (SB) criteria for FH were also performed.

**Results:**

The prevalence of genetically confirmed FH was 38.1% (n = 40) in 105 patients. DLCN categorized 26.7% patients to probable and definite FH while SB identified 17.1% of patients with possible to definite FH. Twenty-five (62.5%) and seventeen (42.5%) patients with pathogenic mutations were undiagnosed according to SB and DLCN criteria. FH variant carriers, especially homozygotes, had significantly higher plasma LDL-C levels. The best LDL-C threshold for genetically confirmed FH was 4.56 mmol/L in the present study.

**Conclusions:**

FH is really a common cause for very young CAD patients (≤ 35 years) with a 38.1% of causative mutations in China and best LDL-C threshold for predicting mutations was 4.56 mmol/L. The underdiagnostic rate of clinical criteria was around 42.5–62.5%, suggesting that the expanded genetic testing could indeed promote the diagnosis of FH.

**Electronic supplementary material:**

The online version of this article (10.1186/s12967-018-1737-7) contains supplementary material, which is available to authorized users.

## Background

Familial hypercholesterolemia (FH), a monogenic autosomal dominant disorder of low-density lipoprotein cholesterol (LDL-C) metabolism, is a worldwide health burden demonstrated by a recent FH Expert Panel [1]. Patients with FH have lifelong elevated levels of low-density lipoprotein (LDL) particles, as well as increased LDL-C arterial deposits, leading to coronary artery disease (CAD), namely myocardial infarction (MI) and angina pectoris [2, 3]. Moreover, untreated FH patients have an increased risk of premature CAD, especially for homozygotes who will develop ASCVD before 20 years old and generally not surviving past 30 years [4, 5].

Currently the diagnosis of FH is commonly performed according to genetic testing and clinical phenotypes. As we well known, FH has been classified into heterozygous and homozygous forms depending on the presence of affected alleles in genes encoding the LDL receptor (LDLR), apolipoprotein B (ApoB), and proprotein convertase subtilisin/kexin type 9 (PCSK9) [1, 2]. Next-generation sequencing has shown that the FH phenotype occasionally results from dominant mutations in *APOE* or *STAP*1 [4, 6]. Additionally, rare variants in *LDLRAP1, LIPA*, and *ABCG5/8* cause a purely autosomal recessive hypercholesterolemia, in which recessive forms have hypercholesterolemia phenotypically similar to FH [7]. However, most of the previous studies concerning genetic testing performed one or three common genes analyses. The recent published Expert Panel suggested that genetic testing is the “gold standard” for FH diagnosis and expand panels could be performed to improve the diagnostic rate [1].

Diagnosis of FH is also based on clinical criteria and the most widely used FH clinical criteria are those of the Simon Broome (SB) Register Group [8] in the United Kingdom and the Dutch Lipid Clinic Network (DLCN) [6, 9]. The prevalence of FH historically estimated to be on the order of 1:500 [1], however with advance in molecular diagnosis, recent data suggested that it could be around 1:200 [10]. The NICE guidelines [6] and recent Expert Panel [1] for the identification and management of FH patients recommend that all patients with clinical features of FH should be offer a genetic testing to confirm their diagnosis. However, many individuals and families with FH are still underdiagnosed and undertreated in most countries, thereby causing a major global public health challenge [6].

The prevalence of CAD has increased and manifested a younger trend, which has becoming an important public health issue. Untreated FH individuals aged 20–39 years were at 100-fold increase in mortality from CAD compared to those of general population [8]. The risk factors for young group are largely uncertain and differ from older patients. Recent data reported that phenotypic diagnosis of FH was relatively common in these high-risk patients [11]. Genetic testing showed that hypercholesterolemic individuals aged < 40 years were more likely to carry an FH-causing mutation than individuals ≥ 40 years [12]. All these data suggested that genetic testing should be made to detect FH in patients with very early-onset CAD and to initiate statin therapy to prevent the development of CAD.

The proportion of DNA-confirmed FH patients hospitalized with very early-onset (≤ 35 years) CAD remains uncertain. To fill these gaps, we aimed to assess the prevalence of genetically confirmed FH in patients with very early-onset CAD and to evaluate the diagnostic performance of FH clinical criteria compared with FH genetic findings.

## Methods

### Study population

From March 2012 to March 2017, a total of 10,275 patients were consecutively recruited from Fuwai Hospital. Patients were defined with CAD when presenting a stenosis ≥ 50% in at least one major coronary artery as previously reported [11]. Very early-onset CAD was defined as clinical CAD occurring by age ≤ 35 years in our study. Patients with very early-onset CAD and plasma LDL-C ≥ 3.4 mmol/L were included. Exclusion criteria were the presence of serious heart failure or arrhythmia, infectious or systematic inflammatory disease, significant hematologic disorders, thyroid dysfunction, and severe liver dysfunction. Patients were also excluded if without information on cholesterol levels or with lipid disorders secondary to renal, thyroid, or liver diseases.

The study protocol complied with the Declaration of Helsinki and was approved by hospital’s ethical review board (FuWai Hospital & National Center for Cardiovascular Diseases, Beijing, China). Informed written consents were obtained from all patients enrolled in this analysis.

### Clinical assessment

After admission, clinical data were collected from physical examination (including xanthomas and corneal arcus) and medical interview including family history, smoking status, alcohol consumption, and past medical history. Body mass index (BMI) was calculated as weight (kg) divided by square of their height (m^2^). Hypertension was defined as repeated blood pressure measurements ≥ 140/90 mmHg for at least three times in different environments or currently taking anti-hypertensive drugs. Diabetes mellitus (DM) was defined as a fasting serum glucose level ≥ 7.0 mmol/L, random glucose ≥ 11.1 mmol/L, glycated hemoglobin > 6.5%, and/or the current use of medication for diabetes.

### Laboratory examinations

Blood samples were obtained from the peripheral veins of all patients after a 12-h overnight fast and were stored at − 80 °C until analysis. Plasma total cholesterol (TC), triglycerides (TG), high-density lipoprotein cholesterol (HDL-C), and LDL-C concentrations were measured using a Hitachi 7150 automated analyser (Hitachi, Japan). Lipoprotein (a) [Lp(a)] levels were assayed by an immunoturbidimetric method [LASAY Lp(a) auto; SHIMA Laboratories] as previously described [8]. LDL-C levels were estimated by correction factors if patients received statin therapy before admission or cannot obtain the highest LDL-C levels according to previous studies [9].

### Clinical diagnostic criteria for FH

Two criteria recommended by international guidelines were widely performed to clinical diagnosis of FH (Additional file 1: Tables S1 and S2). The SB criteria considers a diagnosis of possible FH as TC level > 7.5 mmol/L or LDL-C > 4.9 mmol/L plus a family history of premature CAD. Definite FH diagnosis was defined as aforementioned cholesterol levels and the presence of tendon xanthomas in patient or relatives. The following numerical score was employed in DLCN algorithm: (1) family history of a first-degree relative with known premature CAD (≤ 55 years for men; ≤ 60 years for women, 1 point) and/or with known hypercholesterolemia (1 point) or xanthomas (2 points) or offspring(s) with known hypercholesterolemia (2 point); (2) personal history of premature CAD (ages as above, 2 points) or cerebral/peripheral vascular disease (ages as above, 1 point); (3) xanthomas (6 points) or corneal arcus (4 points); (4) LDL-C > 8.5 mmol/L (8 points), 6.5–8.4 mmol/L (5 points), 5.0–6.4 mmol/L (3 points), or 4.0–4.9 mmol/L (1 point). Finally, a diagnosis of definite FH was considered if the total score was > 8 points, probable if the score was 6–8 points, possible if the score was 3–5 points, and unlikely if the score was < 3 points.

### DNA analysis, variant data and pathogenicity classification

Genomic DNA was prepared from white blood cells following the manufacturer’s standard procedure using a commercially available DNA extraction kit (Tiangen Biotech, Beijing, China). Each DNA sample was purified and quantified with Nanodrop 2000 (Thermo Fisher Scientific, DE). A minimum of 3 mg DNA was used for the indexed Illumina libraries according to manufacturer’s protocol (MyGenostics, Beijing). The final library size 350–450 bp including adapter sequences was selected.

High throughput DNA sequencing was applied for the mutation screening. Briefly, a specific hereditary hypercholesterolemia enrichment panel based on targeted exome capture technology was used to collect of promoters, coding regions, and exon–intron boundaries of 5 genes associated with FH (*LDLR, APOB, PCSK9, APOE,* and *STAP1*) and 4 genes associated with other conditions that have partially overlapping clinical features with FH (*LDLRAP1*, *LIPA*, and *ABCG5/ABCG8*) according to the manufacturer’s description. The exon-enriched DNA libraries were then prepared for high throughput sequencing with the Illumina HiSeq 2000 (Illumina, San Diego, CA) platform. The obtained mean exome coverage was more than 98%, with variants accuracy at more than 99%. Then using the Solexa QA the cutadapt (http://code.google.com/p/cutadapt/), SOAP aligner, BWA, and GATK programs to retrieve and align to identify SNPs and insertions or deletions (InDels). SNPs and InDels were annotated using the exome-assistant program (http://122.228.158.106/exomeassistant). Noncommon variants were defined as a minor allele frequency < 1% in the general population. The potential pathogenicity of rare variants was evaluated the following criteria: (1) reported as pathogenicity by published articles; (2) loss-of-function variants caused by insertions, deletions, point mutations at sites of pre-messenger ribonucleic acid splicing or introducing a stop codon; (3) missense variants predicted to be deleterious by more than two silico prediction algorithms (ClinVar, PathSNP, Sorting Intolerant From Tolerant [SIFT], PolyPhen-2 HumVar, MutationTaster, InterVar, Interpro, SPIDEX, gnomAD); (4) a private database was also performed to evaluate the genetic variants. Finally, the variants were classified as pathogenic (class I), likely pathogenic (class II), and variants of unknown significance (VUS) (class III). Patients with 2 variant alleles were defined as two mutations. Additionally, we performed the Sanger sequencing to validate the novel mutations of target next-generation sequencing as described previously [9].

### Statistical analysis

The data were expressed as the mean ± SD or median (interquartile range [IQR]) for the continuous variables and the number (percentage) for the categorical variables. The Student’s *t* test, one-way analysis of variance, or non-parametric test was used for the comparison between/among groups of continuous parameters as appropriate. The categorical variables were compared using the Chi square test. Receiver operating characteristic (ROC) curve was used to determine the LDL-C threshold value for the prediction of FH mutations. The level of statistical significance was set at p ≤ 0.05. All statistical analysis was performed using IBM SPSS Statistics for Mac version 22.0 (IBM SPSS Statistics, IBM Corporation, Armonk, New York).

## Results

Among 10,275 patients, 105 ones finally met the inclusion criteria and were included in our study. The baseline demographic and clinical characteristics of patients with very early-onset CAD are shown in Table 1. Patients were 94.3% male, with a mean age of 32 years. Mean LDL-C level at admission was 5.77 ± 3.38 mmol/L and 81 patients (77.1%) were receiving statin therapy. Fifty-two patients (49.5%) had a history of MI and 20 (19.0%) had family history of CAD. None of the patients had been diagnosed with FH previously.

**Table 1:** Clinical and laboratory characteristics of all the patients

Characteristics	Total (n = 105)
Age, years	31.69 ± 5.65
Male, n (%)	99 (94.3)
BMI, kg/(m^2^)	29.07 ± 20.14
Family history of premature CAD, n (%)	20 (19.0)
History of MI, n (%)	52 (49.5)
Currently smoking, n (%)	70 (66.7)
Alcohol drinker, n (%)	42 (40.0)
Hypertension, n (%)	46 (43.8)
DM, n (%)	17 (16.2)
Statin, n (%)	81 (77.1)
TG, mmol/L	1.80 ± 0.84
TC, mmol/L	6.62 ± 7.49
HDL-C, mmol/L	0.89 ± 0.26
LDL-C, mmol/L	5.77 ± 3.38
Lp(a), mg/dL	210.40 (65.75–496.06)
Xanthoma, n (%)	12 (11.4)
Mutations, n (%)	40 (38.1)
*LDLR*, n (%)	15 (14.3)
*APOB*, n (%)	7 (6.7)
*PCSK9*, n (%)	2 (1.9)
*STAP1*, n (%)	1 (1.0)
*LDLR* Homozygote, n (%)	4 (4.8)
Two mutations, n (%)	11 (10.5)

Data are expressed as mean ± SD, or n (%). *BMI* body mass index, *CAD* coronary artery disease, *MI* myocardial infarction, *DM* diabetes mellitus, *TG* triglyceride, *TC* total cholesterol, *HDL-C* high-density lipoprotein cholesterol, *LDL-C* low-density lipoprotein cholesterol, *Lp(a)* lipoprotein (a), *APOB* apolipoprotein B, *LDLR* low-density lipoprotein receptor, *PCSK9* proprotein convertase subtilisin/Kexin type 9, *STAP1* signal-transducing adaptor protein 1

As shown in Table 1, the prevalence of FH pathogenic mutations was 40 (38.1%) in 105 patients, corresponding to about a total carrier frequency of 1:3 in patients with very early-onset CAD. There were 15 patients (14.3%) with *LDLR* mutations, 7 (6.7%) with *APOB* gene mutations, 2 (1.9%) with *PCSK9* gene mutation and 1 (1.0%) with *STAP1* mutation. Four patients (4.8%) were homozygote for variants in *LDLR*. Eleven FH mutation-positive patients (10.5%) were found to have 2 variant alleles. Additionally, 6 had a heterozygous *LIPA* gene mutation, 4 and 7 of the patients had mutations in *LDLRAP1* and *ABCG5/8* genes, respectively. The detailed list of mutations is reported in Additional file 1: Tables S3 and S4.

When we stratified very early-onset CAD patients on the basis of DLCN score, the LDL-C levels augmented along with the DLCN score increased (Additional file 1: Table S5). Interestingly, patients with score 6–8 had the highest prevalence of FH genetic mutations (91.7%). The distribution of *LDLR* homozygote was concentrated in DLCN score > 8. Similarly, the distribution of two mutations was also centralized in DLCN > 8. The plasma LDL-C levels according to genotype were shown in Additional file 1: Table S6 and Fig. S1. The mean LDL-C concentrations in patients of *LDLR* homozygote were the highest (13.88 ± 4.72 mmol/L). Patients of 2 mutant alleles (8.21 ± 3.23, *p *= 0.0025) and *LDLR* (7.46 ± 4.93, *p *= 0.0086) mutations had significantly higher LDL-C levels compared with FH mutation-negative patients. There was no significant difference in plasma LDL-C levels among *APOB, PCSK9* and *STAP1* variants although all of them were higher than LDL-C levels of FH mutation-negative patients.

The clinical and biochemical characteristics of patients with very early-onset CAD according to FH genetic mutations were presented in Table 2. Compared with mutation-negative patients, mutation-positive patients were younger (29.90 ± 7.57 vs 32.78 ± 3.70, *p *= 0.029), and had higher LDL-C (7.65 ± 4.49 vs 4.61 ± 1.63, *p *< 0.001), and Lp(a) (327.09 [107.75–532.10] vs 110.59 [55.03–404.98], *p *= 0.033) concentrations. Meanwhile, mutation-positive patients had significant higher prevalence of xanthoma (25.0% vs 3.1%, *p *= 0.001). There were no differences between groups regarding TC and HDL-C levels. Statin treatment did not differ between patients with and without FH genetic mutations. There were no differences between the groups regarding the presence of previous MI or family history of premature CAD.

**Table 2:** Biochemical and clinical characteristics of patients with different mutations

Characteristics	FH mutation (n = 40)	No FH mutation (n = 65)	p value
Age, years	29.9 ± 7.57	32.78 ± 3.70	0.029
Male, n (%)	35 (87.5)	64 (98.5)	0.019
BMI, kg/(m^2^)	25.16 ± 5.06	31.35 ± 24.85	0.133
Family history of premature CAD, n (%)	9 (22.5)	11 (16.9)	0.480
History of MI, n (%)	21 (52.5)	31 (47.7)	0.632
Currently smoking, n (%)	20 (50.0)	50 (76.9)	0.004
Alcohol drinker, n (%)	10 (25.0)	32 (49.2)	0.014
Hypertension, n (%)	9 (25.0)	37 (56.9)	0.001
DM, n (%)	4 (10.0)	13 (20.0)	0.177
Statin, n (%)	29 (72.5)	52 (80.0)	0.374
TG, mmol/L	1.95 ± 0.89	1.57 ± 0.7	0.025
TC, mmol/L	7.78 ± 4.01	6.14 ± 8.97	0.410
HDL-C, mmol/L	0.86 ± 0.27	0.92 ± 0.26	0.249
LDL-C, mmol/L	7.56 ± 4.49	4.61 ± 1.63	< 0.001
Lp(a), mg/dL	327.09 (107.75–532.10)	110.59 (55.03–404.98)	0.033
Xanthoma, n (%)	10 (25.0)	2 (3.1)	0.001

Data are expressed as mean ± SD, or n (%). *FH* familial hypercholesterolemia, *BMI* body mass index, *CAD* coronary artery disease, *MI* myocardial infarction, *DM* diabetes mellitus, *TG* triglyceride, *TC* total cholesterol, *HDL-C* high-density lipoprotein cholesterol, *LDL-C* low-density lipoprotein cholesterol

In 105 patients with very early-onset CAD, there were 10 (9.5%) patients with definite FH, 8 (7.6%) with possible FH, and 87 (82.9%) with unlikely FH by SB criteria. DLCN criteria classified 28 patients (26.7%) with probable or definite FH, of which 16 patients (15.2%) had definite FH, 12 patients (11.4%) had probable FH and 49 patients (46.7%) met the criteria for possible FH. Apart from 40 patients (38.1%) with pathogenic or likely pathogenic FH mutations, genetic testing also revealed 15 (14.3%) of the patients had FH VUS mutations and 50 patients (47.6%) had no genetic mutation associated with FH.

When DLCN criteria were applied with the genetic evaluation for FH, 12 (30.0%) patients met criteria for definite and 11 (27.5%) met criteria for probable FH diagnosis (Table 3 and Additional file 1: Fig. S2). After applying SB criteria, there were 7 (17.5%) and 8 (20.0%) patients classified as ‘possible’ and ‘definite’ FH respectively. Seventeen FH mutation-positive patients (42.5%) failed to be confirmed by DLCN criteria, and 25 (62.5%) were not diagnosed FH according to SB criteria. Whereas among FH mutation-negative patients (n = 65), there were 2 (3.1%) ‘definite’, and 1 (1.5%) ‘possible’ FH diagnoses according to SB criteria. Moreover, 5 patients (7.7%) fulfilling DLCN criteria for FH exhibited no FH mutation.

**Table 3:** Clinical scores of patients with or without FH mutation

	FH mutation (n = 40)	No FH mutation (n = 65)	p value
Simon Broome criteria			
Unlikely FH	25 (62.5%)	62 (95.4%)	
Possible FH	7 (17.5%)	1 (1.5%)	
Definite FH	8 (20.0%)	2 (3.1%)	
(Possible and definite)	15 (37.5%)	3 (4.6%)	< 0.001
Dutch Lipid Clinic criteria			
Unlikely FH	3 (7.5%)	25 (38.5%)	
Possible FH	14 (35.0%)	35 (53.8%)	
Probable FH	11 (27.5%)	1 (1.5%)	
Definite FH	12 (30.0%)	4 (6.2%)	
(Probable and definite)	23 (57.5%)	5 (7.7%)	< 0.001

Data are expressed as n (%). *FH* familial hypercholesterolemia

**Fig. 1:**
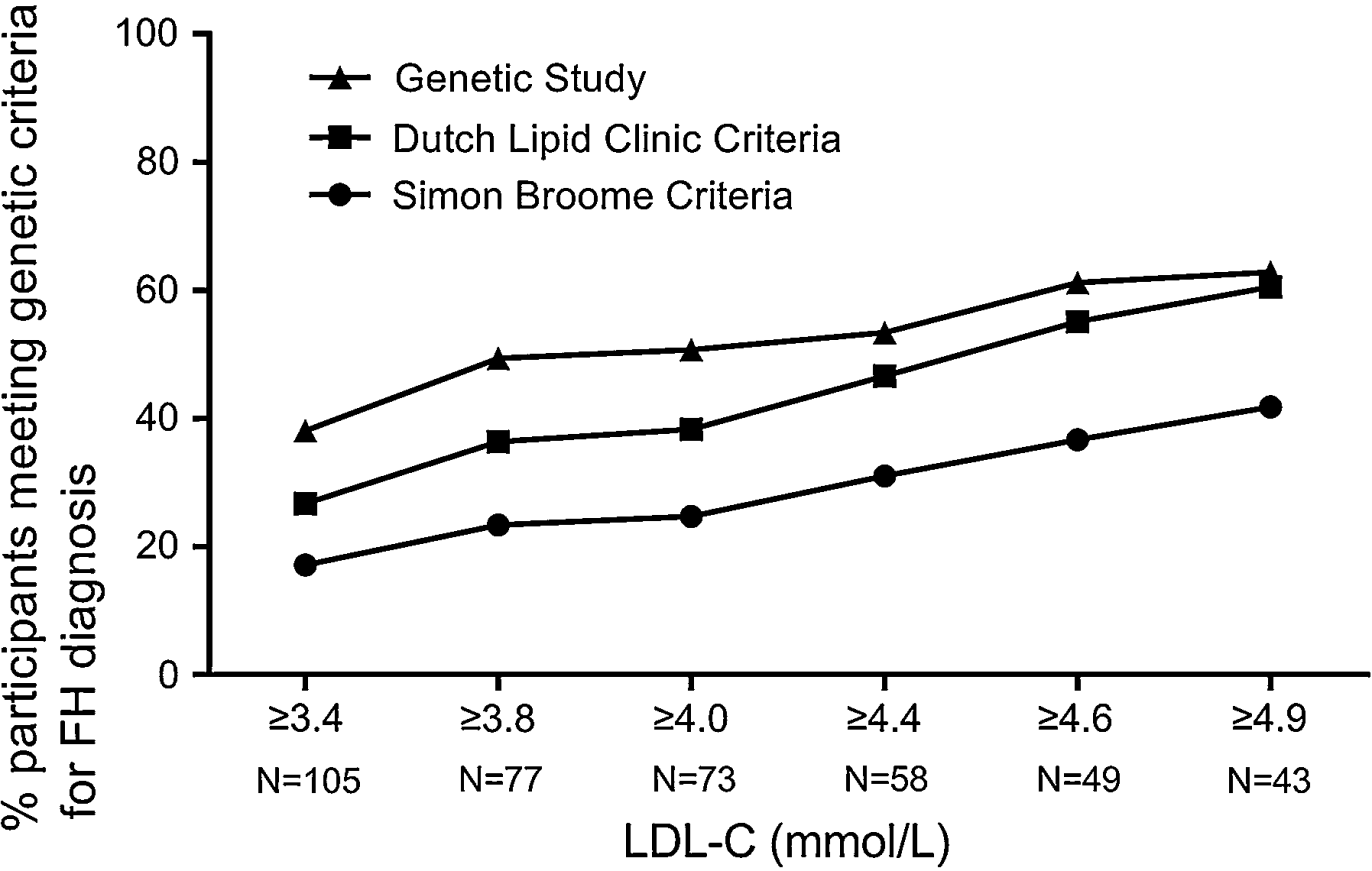
Diagnostic rate of FH by increasing LDL-C levels. *LDL-C* low-density lipoprotein cholesterol

To assess the efficacy of initial LDL-C concentrations to diagnose of FH, we applied different LDL-C levels according to different guidelines or recommendations (Fig. 1, Additional file 1: Table S7). The positive detection rate of FH increased with initial LDL-C concentrations in three groups by SB criteria, DLCN criteria and genetic testing. Among all the different initial LDL-C concentration groups, more FH patients were diagnosed by genetic study. In very early-onset CAD patients with LDL-C higher than 4.9 mmol/L, the prevalence of FH was similar by genetic study and DLCN criteria (62.8% vs 60.5%). ROC curve was used to find the best LDL-C threshold values to predict molecular diagnoses of FH in patients with very early-onset CAD (Additional file 1: Fig. S3). The area under curve (AUC) prompted that the LDL-C had properly discriminatory power for predicting FH-positive mutations (AUC = 0.803, 95% confidence interval: 0.716–8.889) and the optimal cut-off value was 4.56 mmol/L with a sensitivity of 77.5% and specificity of 70.8%.

## Discussion

Underdiagnosis and undertreatment of FH is still a clinical challenge, therefore representing a major global public health burden [1, 6]. In the present study, we for the first time performed a genetic analysis of FH causative mutations in consecutive 105 very early-onset CAD patients (≤ 35 years), with plasma LDL-C ≥ 3.4 mmol/L. Our study had three major findings: (1) the prevalence of genetically confirmed FH in these patients was 38.1%, suggesting that one of three Chinese patients with very early-onset CAD might be FH; (2) clinical criteria had a high rate of underdiagnosis and might not accurately identify FH patients among these with very early-onset CAD; (3) LDL-C values ≥ 4.56 mmol/L had the best tradeoff between sensitivity and specificity to diagnose a mutation in patients with very early-onset CAD.

Although the prevalence rate of CAD in very young individuals is low, it can cause devastating consequences. During the past decades, a large amount of studies were applied to assess the potential risk factors and finally determined heavy smoking, BMI, hypertension, and family history of CAD [13, 14]. However, all these are traditional risk factors for common CAD patients. With the development of genetic testing, FH were gradually recognized and widely diagnosed. Previous studies showed that maximal coronary flow and flow reserve were significantly lower in young patients with FH than in matched healthy control participants, which supported the concept that the abnormal serum lipid profile is associated with abnormal coronary flow response [15, 16]. Clinically, compared to hypercholesterolemic patients aged ≥ 40 years, a twofold higher FH mutation detection rate was found in individuals aged < 40 years [12]. Moreover, people younger than 35 years are in reproductive age and early detection of FH may be beneficial to reproductive options [1]. Therefore, it is critical to early identify FH in patients with very early-onset CAD.

There might be several ways to improve the early identification of FH, for example, genetic screening young patients with CAD. Koivisto et al. [17] reported that the prevalence of DNA-conformed HeFH in 150 patient with MI less than 45 years was 9%. However, in a sample of French Canadian men aged < 45 years who underwent coronary angiography for chest pain, the detection rate was 16.4% [18]. Using a molecular diagnosis of FH, Rubba et al. [19] identified 82% causative mutations of *LDLR, APOB*, and *PCSK9* in young patients with a family history of hypercholesteremia or premature CAD and LDL-C ≥ 4.9 mmol/L. As to younger patients, Rallidis et al. [20] recruited 320 patients in Greece with MI ≤ 35 years and identified 20.3% with definite/probable FH using the DLCN criteria. Unfortunately, this study did not show a genetic testing result. Our study performed both clinical and genetic assessment to diagnose FH in patients with very early-onset CAD and found that FH-causing mutations were estimated to occur in 1:3 in these patients. Although discrepancies among the above studies may be due to differences in spectrum of gene mutation, study design, and patient characteristics, further study may be needed to get more information regarding FH in very young patients with CAD.

Expanded genetic analysis has also been recommended by recent FH Export Panel for further improving the FH diagnostic rate [1]. Previous studies genotyped for only *LDLR* and showed a prevalence of no more than 9% in young patients with CAD [17, 21]. Young patients with chest pain evaluating for 2 causative mutations showed 16.4% prevalence of FH [18]. The occurrence of FH genetic mutation was reported in 77% of the young unrelated patients with LDL-C ≥ 4.9 mmol/L and family history of hypercholesteremia or premature CAD [19]. Amor-Salamanca et al. [22] found the prevalence of genetically confirmed FH in 106 patients with acute coronary syndrome, age ≤ 65 years, and LDL-C ≥ 160 mg/dL was approximately 9% in a European cohort by testing 7 FH-related genes. In our study, 105 patients with CAD, age ≤ 35 years, and LDL-C ≥ 3.4 mmol/L were evaluated for 9 genes (*LDLR, APOB, PCSK9, APOE, STAP1, LIPA, LDLRAP1, ABCG5/8*) for FH diagnosis. The important finding was that FH-causing mutations were estimated to occur in 1:3 in these patients, which was the highest rate of FH diagnosis up to date. The difference in methodology and include criteria could partly explain the differences found between studies and should be considered when interpreting these results. Nevertheless, all the previous studies pointed out the importance of early recognition of FH by genetic testing in young patients with CAD, especially these ≤ 35 years.

The diagnosis of FH using clinical criteria is usually the first step in identifying possible FH patients. However, one of the main findings of our study suggested that expanded FH genetic testing provided more precise and unambiguous diagnosis than clinical criteria. Accurately, 42.5–62.5% patients with genetically confirmed FH mutation were not detected by clinical criteria, whereas 4.6–7.7% patients fulfilling clinical criteria for FH exhibited no FH mutation. This suggested that clinical criteria might be of limited utility when applied to patients with very early-onset CAD in the absence of genetic testing, as aligned with recent publications [1, 22]. Therefore, it was critical to establish a most optimal threshold for LDL-C concentration to discriminate FH mutation-positive patients in this subgroup with very early-onset CAD. The Copenhagen General Population Study genotyped *LDLR* and *AOPB* in general population and concluded that 4.4 mmol/L was the most optimal cutoff value for all ages [10]. Silva et al. [23] tested six FH-related genes in patients with LDL-C level above or equal to 5.4 mmol/L in Brazil and found that the LDL-C ≥ 5.96 mmol/L cutoff was identified as the best value in the age groups ≤ 40 years. Using molecular techniques for *LDLR* and *APOB*, Mickiewicz et al. [12] demonstrated that LDL-C thresholds for FH were 5.79 mmol/L in individuals aged < 40 with baseline LDL-C level ≥ 4.9 mmol/L. In present study, 4.56 mmol/L had the best tradeoff between sensitivity and specificity to diagnose a FH genetically confirmed mutation. We thought the lower cutoff value found in our study was partly related to the LDL-C threshold used (3.4 mmol/L), which was designed to include all young patients with abnormal LDL-C levels. Moreover, the LDL-C levels in mutation-positive FH patients vary according to country and ethnicity. Precisely, Asian appeared to have lower LDL-C levels than Europeans [24–26]. Considering the Asian background, it was rational to propose 4.56 mmol/L as an ideal cut point for LDL-C concentration in our study. Nevertheless, we concluded that all patients ≤ 35 years with CAD should be qualified for genetic testing by reason that younger individuals are likely to benefit most from the early diagnosis and statin treatment. However, this threshold needs to be characterized in larger population.

It was noted that, Lp(a) levels were higher in patients with FH mutations than those without, indicating that Lp(a) might be an independent predictor of very early-onset CAD. Our results were in line with previous findings that risk of CAD is higher in FH mutation-positive patients with an Lp(a) level > 50 mg/dL compared with nonaffected patients [27, 28]. Additionally, in this study patients who were positive for FH mutations or diagnosed by DLCN criteria were younger and had relatively higher LDL-C concentrations, suggesting that younger patients with CAD have the higher possibility of FH. Notably, there were two patients with xanthoma did not exhibit any FH mutation, indicating the difference between clinical features and genetic testing. Finally, we noticed that, 4.6–7.7% fulfilling clinical criteria patients in this study had no identifiable genetic mutations. Possible determinants to explain the phenotype in these patients included: (1) other genes associated with hypercholesterolemia or even undiscovered gene at present [29]; (2) polygenic variants instead of monogenic disorder of hypercholesterolemia; (3) variants affecting cholesterol metabolism through non-Mendelian inheritance, like mitochondrial or epigenetic; (4) environmental factors acquired response instead of inheritance.

When interpreting the results of this study, several limitations need to be considered. First, in present study plasma LDL-C of patients on lipid-lowering medications with their pretreatment LDL-C unavailable were adjusted by a relative correction factor, which might be inaccurate since the heterogeneity in individual response or mutation status. Second, cascade genetic testing was not applied in our study due to hardly collection of blood sample from relatives of included patient. Furthermore, a limitation of our study is the relatively small sample size. However, young CAD patients are relatively rare. Finally, owing to its unicentral design, our results should be replicated in large prospective studies. Nonetheless, our study was the first study on expanded genetic analysis for identifying the FH mutations in CAD patients with less than 35 years. Further study in a larger population could refine the treatment of FH patients in the future.

## Conclusion

In conclusions, in the present study on Chinese CAD patients with ≤ 35 years, we firstly suggested that the prevalence of mutation-positive FH was high (38.1%) and about 1 of 3 patients were associated with FH. More importantly, the traditional clinical criteria showed limited mutation detection power and low specificities in Chinese FH patients with very early-onset CAD, in whom the best LDL-C threshold for genetically confirmed FH was 4.56 mmol/L.

## Additional file

**Additional file 1: Table S1.** Simon Broome diagnostic criteria for familial hypercholesterolemia. **Table S2.** Dutch Lipid Clinic Network Clinical Criteria for familial hypercholesterolemia. **Table S3.** Summary of pathogenic/likely pathogenic mutations in CAD patients. **Table S4.** Summary of variants of unknown significance in CAD patients. **Table S5.** Biochemical and clinical characteristics of patients in relation to DLCN Scores. **Table S6.** Plasma LDL-C levels stratified by genetic mutations in the patients with very early-onset CAD. **Figure S1.** Plasma LDL-C levels stratified by genetic mutations in the patients with very early-onset CAD. **Figure S2.** Percentage participants with CAD meeting clinical and genetic criteria for FH diagnosis. **Table S7.** Percentage participants with early-onset CAD meeting clinical and genetic criteria for FH diagnosis based on different initial LDL-C levels. **Figure S3.** Receiver operating characteristic curves of LDL-C (n = 105).

## References

[1] Sturm AC, Knowles JW, Gidding SS, Ahmad ZS, Ahmed CD, Ballantyne CM (2018). Clinical genetic testing for familial hypercholesterolemia: JACC scientific expert panel. J Am Coll Cardiol.

[2] Marks D, Thorogood M, Neil HA, Humphries SE (2003). A review on the diagnosis, natural history, and treatment of familial hypercholesterolaemia. Atherosclerosis.

[3] Li JJ, Li S, Zhu CG, Wu NQ, Zhang Y, Guo YL (2017). Familial hypercholesterolemia phenotype in Chinese patients undergoing coronary angiography. Arterioscler Thromb Vasc Biol.

[4] Cuchel M, Bruckert E, Ginsberg HN, Raal FJ, Santos RD, Hegele RA (2014). Homozygous familial hypercholesterolaemia: new insights and guidance for clinicians to improve detection and clinical management. A position paper from the Consensus Panel on Familial Hypercholesterolaemia of the European Atherosclerosis Society. Eur Heart J.

[5] Strong JP, Malcom GT, McMahan CA, Tracy RE, Newman WP, Herderick EE (1999). Prevalence and extent of atherosclerosis in adolescents and young adults: implications for prevention from the Pathobiological Determinants of Atherosclerosis in Youth Study. JAMA.

[6] Nordestgaard BG, Chapman MJ, Humphries SE, Ginsberg HN, Masana L, Descamps OS (2013). Familial hypercholesterolaemia is underdiagnosed and undertreated in the general population: guidance for clinicians to prevent coronary heart disease: consensus statement of the European Atherosclerosis Society. Eur Heart J.

[7] Brautbar A, Leary E, Rasmussen K, Wilson DP, Steiner RD, Virani S (2015). Genetics of familial hypercholesterolemia. Curr Atheroscler Rep.

[8] Scientific Steering Committee on behalf of the Simon Broome Register Group (1991). Risk of fatal coronary heart disease in familial hypercholesterolaemia. BMJ.

[9] Li S, Zhang Y, Zhu CG, Guo YL, Wu NQ, Gao Y (2016). Identification of familial hypercholesterolemia in patients with myocardial infarction: a Chinese cohort study. J Clin Lipidol.

[10] Benn M, Watts GF, Tybjaerg-Hansen A, Nordestgaard BG (2016). Mutations causative of familial hypercholesterolaemia: screening of 98 098 individuals from the Copenhagen General Population Study estimated a prevalence of 1 in 217. Eur Heart J.

[11] Li S, Zhang HW, Guo YL, Wu NQ, Zhu CG, Zhao X (2018). Familial hypercholesterolemia in very young myocardial infarction. Sci Rep.

[12] Mickiewicz A, Chmara M, Futema M, Fijalkowski M, Chlebus K, Galaska R (2016). Efficacy of clinical diagnostic criteria for familial hypercholesterolemia genetic testing in Poland. Atherosclerosis.

[13] Berenson GS, Srinivasan SR, Bao W, Newman WP, Tracy RE, Wattigney WA (1998). Association between multiple cardiovascular risk factors and atherosclerosis in children and young adults. The Bogalusa Heart Study. N Engl J Med.

[14] Allen J, Markovitz J, Jacobs DR, Knox SS (2001). Social support and health behavior in hostile black and white men and women in CARDIA. Coronary Artery Risk Development in Young Adults. Psychosom Med.

[15] Pitkanen OP, Raitakari OT, Niinikoski H (1996). Coronary flow reserve is impaired in young men with familial hypercholesterolemia. J Am Coll Cardiol.

[16] Dayanikli F, Grambow D, Muzik O, Mosca L, Rubenfire M, Schwaiger M (1994). Early detection of abnormal coronary flow reserve in asymptomatic men at high risk for coronary artery disease using positron emission tomography. Circulation.

[17] Koivisto UM, Hamalainen L, Taskinen MR, Kettunen K, Kontula K (1993). Prevalence of familial hypercholesterolemia among young north Karelian patients with coronary heart disease: a study based on diagnosis by polymerase chain reaction. J Lipid Res.

[18] Gaudet D, Vohl MC, Julien P, Tremblay G, Perron P, Gagne C (1998). Relative contribution of low-density lipoprotein receptor and lipoprotein lipase gene mutations to angiographically assessed coronary artery disease among French Canadians. Am J Cardiol.

[19] Rubba P, Gentile M, Marotta G, Iannuzzi A, Sodano M, De Simone B (2017). Causative mutations and premature cardiovascular disease in patients with heterozygous familial hypercholesterolaemia. Eur J Prev Cardiol.

[20] Rallidis LS, Triantafyllis AS, Tsirebolos G, Katsaras D, Rallidi M, Moutsatsou P (2016). Prevalence of heterozygous familial hypercholesterolaemia and its impact on long-term prognosis in patients with very early ST-segment elevation myocardial infarction in the era of statins. Atherosclerosis.

[21] Wald DS, Bangash FA, Bestwick JP (2015). Prevalence of DNA-confirmed familial hypercholesterolaemia in young patients with myocardial infarction. Eur J Intern Med.

[22] Amor-Salamanca A, Castillo S, Gonzalez-Vioque E, Dominguez F, Quintana L, Lluis-Ganella C (2017). Genetically confirmed familial hypercholesterolemia in patients with acute coronary syndrome. J Am Coll Cardiol.

[23] Silva PRS, Jannes CE, Oliveira TGM, Miname MH, Rocha VZ, Chacra AP (2017). Evaluation of clinical and laboratory parameters used in the identification of index cases for genetic screening of familial hypercholesterolemia in Brazil. Atherosclerosis.

[24] Civeira F, Ros E, Jarauta E, Plana N, Zambon D, Puzo J (2008). Comparison of genetic versus clinical diagnosis in familial hypercholesterolemia. Am J Cardiol.

[25] Chiou KR, Charng MJ (2010). Detection of mutations and large rearrangements of the low-density lipoprotein receptor gene in Taiwanese patients with familial hypercholesterolemia. Am J Cardiol.

[26] Hu M, Lan W, Lam CW, Mak YT, Pang CP, Tomlinson B (2013). Heterozygous familial hypercholesterolemia in Hong Kong Chinese. Study of 252 cases. Int J Cardiol.

[27] Li S, Wu NQ, Zhu CG, Zhang Y, Guo YL, Gao Y (2017). Significance of lipoprotein(a) levels in familial hypercholesterolemia and coronary artery disease. Atherosclerosis.

[28] Alonso R, Andres E, Mata N, Fuentes-Jiménez F, Badimón L, López-Miranda J (2014). Lipoprotein(a) levels in familial hypercholesterolemia: an important predictor of cardiovascular disease independent of the type of LDL receptor mutation. J Am Coll Cardiol.

[29] Futema M, Plagnol V, Li K, Whittall RA, Neil HA, Seed M (2014). Whole exome sequencing of familial hypercholesterolaemia patients negative for LDLR/APOB/PCSK9 mutations. J Med Genet.

