# Supplementary material for: Application of expanded genetic analysis in the diagnosis of familial hypercholesterolemia in patients with very early-onset coronary artery disease
Source: J Transl Med. 2018 Dec 10;16:345. doi: 10.1186/s12967-018-1737-7 (PMC6288904; doi:10.1186/s12967-018-1737-7)
Supplement: Supplementary file 1 — Additional file 1: Table S1. Simon Broome diagnostic criteria for familial hypercholesterolemia. Table S2. Dutch Lipid Clinic Network Clinical Criteria for familial hypercholesterolemia. Table S3. Summary of pathogenic/likely pathogenic mutations in CAD patients. Table S4. Summary of variants of unknown significance in CAD patients. Table S5. Biochemical and clinical characteristics of patients in relation to DLCN Scores. Table S6. Plasma LDL-C levels stratified by genetic mutations in the patients with very early-onset CAD. Figure S1. Plasma LDL-C levels stratified by genetic mutations in the patients with very early-onset CAD. Figure S2. Percentage participants with CAD meeting clinical and genetic criteria for FH diagnosis. Table S7. Percentage participants with early-onset CAD meeting clinical and genetic criteria for FH diagnosis based on different initial LDL-C levels. Figure S3. Receiver operating characteristic curves of LDL-C (n = 105). [file 12967_2018_1737_MOESM1_ESM.pdf]

Additional file

Application of expanded genetic analysis in the diagnosis of familial hypercholesterolemia in patients with very early-onset coronary artery disease

Ye-Xuan Cao[1*], Na-Qiong Wu [1*], Di Sun[1], Hui-Hui Liu[1], Jing-Lu Jin[1], Sha Li,[1] Yuan-Lin Guo[1], Cheng-Gang Zhu[1], Qiu-Ting Dong[1], Geng Liu[1], Qian Dong, [1] Jian-Jun Li[1]

* The first two authors contributed equally.

Table S1. Simon Broome diagnostic criteria for familial hypercholesterolemia

**Diagnosis**	**Criteria**
Definite FH	Cholesterol >7.5 mmol/L or LDL-cholesterol >4.9 mmol/L in adult
	Cholesterol >6.7 mmol/L or LDL-cholesterol >4.0 mmol/L in a child under 16 years of age
	PLUS
	Tendon xanthomas in patient or a 1st degree relative (parent, sibling, child), or in a 2nd degree relative (grand parent, uncle, aunt)
	OR
	DNA based evidence of a functional *LDLR, PCSK9* and *APOB* mutation
Probable FH	Cholesterol >7.5 mmol/L or LDL-cholesterol >4.9 mmol/L in adult
	Cholesterol >6.7 mmol/L or LDL-cholesterol >4.0 mmol/L in a child under 16 years of age
	PLUS
	Family History of myocardial infarction (MI) before 50 years of age in a 2nd degree relative or below age 60 in a 1st degree relative
	OR
	Family history of raised total cholesterol - >7.5 mmol/L in adult 1st or 2nd degree relative or >6.7 mmol/L in a child or sibling aged <16 years

Table S2. Dutch Lipid Clinic Network Clinical Criteria for familial hypercholesterolemia

Criteria	Points
Family history	
First-degree relative with known premature (men: <55 years; women: <60 years) coronary artery disease or vascular disease, or first-degree relative with known LDL-C level above the 95th percentile by age, gender for country	1
First-degree relative with tendinous xanthomata and/or arcus cornealis, or children aged less than 18 years with LDL-C level above the 95th percentile by age, gender for country	2
Clinical history	
Patient with premature (men: <55 years; women: <60 years) coronary artery disease	2
Patient with premature (men: <55 years; women: <60 years) cerebrovascular or peripheral vascular disease	1
Physical examination	
Tendinous xanthomata	6
Arcus cornealis prior to age 45 years	4
LDL-C levels	
LDL-C ≥8.5 mmol/l (~330 mg/dl)	8
LDL-C 6.5–8.4 mmol/l (~250–329 mg/dl)	5
LDL-C 5.0–6.4 mmol/l (~190–249 mg/dl)	3
LDL-C 4.0–4.9 mmol/l (~155–189 mg/dl)	1
DNA analysis	
Causative mutation in the *LDLR, ApoB* or *PCSK9* gene	8

>8 points Definite FH

6–8 points Probable FH

3–5 points Possible FH

0–2 points Unlikely FH

Table S3. Summary of pathogenic/likely pathogenic mutations in CAD patients

Gene	Nucleotide change	Effect on protein	PMID/Novel	No. of patient
LDLR	c.1158delC	p.Asp386fs	Novel	1
LDLR	c.1474G>A	p.Asp492Asn	9763532	1
LDLR	c.1724T>C	p.Leu575Pro	Novel	1
LDLR	c.1747C>T	p.His583Tyr	22698793	1
LDLR	c.1765G>A	p.Asp589Asn	16250003	1
LDLR	c.1A>T	p.Met1Leu	8831933	1
LDLR	c.2026G>C	p.Gly676Arg	26892515	1
LDLR	c.2389G>A	p.Val797Met	23375686	1
LDLR	c.313+3A>T	-	Novel	1
LDLR	c.510delC	p.Asp172Thrfs*34	22881376	1
LDLR	c.510delC	p.Asp170fs	Novel	1
LDLR	c.532G>T	p.Asp178Tyr	16389549	1
LDLR	c.670G>T	p.Asp224Tyr	Novel	1
LDLR	c.694+4T>G	-	Novel	1
LDLR	c.971G>A	p.Gly324Asp	Novel	1
LDLR homo	c.974G>A	p.Cys325Tyr	21865347	1
LDLR homo	c.1879G>A	p.Ala627Thr	23375686	1
LDLR homo	c.1448G>A	p.Trp483Ter	7903864	1
LDLR homo	c.1206delC	p.Phe403Serfs*10	15241806	1
APOB	c.1594C>T	p.Arg532Trp	Novel	1
APOB	c.6110T>C	p.Ile2037Thr	Novel	1
APOB	c.7223C>T	p.Ser2408Phe	Novel	1
APOB	c.8267G>T	p.Gly2756Val	Novel	1
APOB	c.8462C>T	p.Pro2821Leu	18710658	1
APOB	c.889C>T	p.Arg297Cys	Novel	1
APOB	c.9164A>G	p.Asn3055Ser	Novel	1
PCSK9	c.10G>A	p.Val4Ile	17316651	1
PCSK9	c.644G>A	p.Arg215His	24404629	1
STAP1	c.596A>G	p.Asn199Ser	Novel	1
LDLR, LDLR	c.665G>T; 2054C>T	p.Cys222Phe; p.Pro685Leu	25741868; 1830890	1
LDLR, LDLR	c.292G>A;c.1864G>A	p.Gly98Ser; p.Asp622Asn	16250003; 15701167	1
LDLR, LDLR	c.1432G>A; c.2054C>T	p.Gly478Arg; p.Pro685Leu	23064986; 23155708	1
LDLR, LDLR	c.665G>T; c.1747C>T	p.Cys222Phe; p.His583Thr	2574186; 7903864	1
LDLR, LDLR	c.769C>T; c.1765G>A	p.Arg257Trp; p.Asp589Asn	11462246; 16250003	1
LDLR, LDLR	c.1885_1886insCA; c.1889G>C	p.Phe629Serfs*37; p.Ser630Tyr	Novel; Novel	1
LDLR, LDLR	c.1884_1885insGA; c.1888_1889insAC	p.Phe629Aspfs*37; p.Ser630Asnfs*36	Novel; Novel	1

LDLR, LDLR	c.2336_2337delGA; c.2337_2338insTTTT;	p.Gly779Glufs*2; p.Gly779fs;	Novel; Novel	1
LDLR, APOB	*LDLR* c.682G>T; *APOB* c.2870T>C	*LDLR* p.Glu228Ter; *APOB* p.Ile957Thr	19843101; Novel	1
LDLR, APOB	*LDLR* c.670G>T; *APOB* c.10748A>T	*LDLR* p.Asp224Tyr; *APOB* p.His3583Leu	Novel; Novel	1
LDLR, PCSK9	*LDLR* c.1879G>A; *PCSK9* c.626C>T	*LDLR* p.Ala627Thr; *PCSK9* p.Pro209Leu	23375686; Novel	1

Table S4. Summary of variants of unknown significance in CAD patients.

Gene	Nucleotide change	Effect on protein
APOB	c.10835A>G	p.Ala3612Gly
APOB	c.12016G>A	p.Val4006Ile
APOB	c.1342G>A	p.Ala448Thr
APOB	c.13663G>A	p.Ala4555Thr
APOB	c.288G>T	p.Glyln96His
APOB	c.4163G>A	p.Arg1388His
APOB	c.4556A>G	p.Asn1519Ser
APOB	c.7331G>A	p.Arg2444His
APOB	c.7565G>A	p.Arg2522Gln
APOB	c.7724A>T	p.Lys2575Ile
APOB	c.7729A>C	p.Met2577Leu
PCSK9	c.1487G>A	p.Arg496Gln
PCSK9	c.1954A>G	p.Asn652Ala
APOE	c.149G>A	p.Arg50His

Table S5. Biochemical and clinical characteristics of patients in relation to DLCN Scores.

**All (N=105)**	**DLCN <3 N=28**	**DLCN3-5 N=49**	**DLCN 6-8 N=12**	**DLCN>8 N=16**	**P for trend**
Age, years	32.64 ± 3.08	33.14 ± 4.03	32.25 ± 3.62	25.13 ± 9.37	0.001
Male, n (%)	28 (100)	48 (98.0)	12 (100)	11 (68.8)	0.097
BMI, kg/(m^2)	34.87 ± 37.65	28.56 ± 3.87	26.76 ± 3.46	21.71±5.83	0.212
Family history of premature CAD, n (%)	0 (0)	10 (20.4)	6 (50.0)	4 (25.0)	0.002
History of MI, n (%)	17 (60.7)	23 (46.9)	6 (50.0)	6 (37.5)	0.483
Currently smoking, n (%)	21 (75.0)	38 (77.6)	7 (58.3)	4 (25.0)	0.001
Alcohol drinker, n (%)	13 (61.9)	17 (38.9)	3 (25.0)	3 (18.8)	0.038
Hypertension, n (%)	12 (42.9)	26 (51.3)	6 (50.0)	2 (12.5)	0.051
DM, n (%)	7 (25.0)	8 (16.3)	2 (16.7)	0 (0)	0.196
Statin, n (%)	21 (75.0)	39 (79.6)	9 (75.0)	12 (75.0)	0.958
TG, mmol/L	1.70 ± 0.68	2.03 ± 0.89	1.63 ± 0.56	1.47 ± 0.98	0.070
TC, mmol/L	3.97 ± 0.98	6.60 ± 10.29	6.22 ± 1.52	11.41 ± 3.95	0.016
HDL-C, mmol/L	0.95 ± 0.33	0.92 ± 0.20	0.87 ± 0.30	0.73 ± 0.23	0.053
LDL-C, mmol/L	3.66 ± 0.16	4.7 ± 0.68	6.78 ± 0.97	12.07±4.71	<0.001
Xanthoma, n (%)	0 (0)	0 (0)	0 (0)	12 (75)	<0.001
% of mutation	3 (10.7)	14 (28.6%)	11(91.7%)	9 (56.3)	
LDLR, n (%)	1 (3.6)	5 (10.2)	6 (50.0)	3 (18.8)	<0.001
APOB, n (%)	2 (7.1)	4 (8.2)	1 (8.3)	0 (0)	0.107
PCSK9, n (%)	0 (0)	1 (2.0)	1 (8.3)	0 (0)	0.001
STAP1, n (%)	0 (0)	1 (2.0)	0 (0)	0 (0)	0.001
LDLR Homozygote, n (%)	0 (0)	0 (0)	0 (0)	4 (25.0)	0.001
Two mutations, n (%)	0 (0)	3 (6.1)	3 (25.0)	5 (56.3)	0.001

Data are expressed as mean ± SD, or n (%). BMI: body mass index; CAD: coronary artery disease; MI: myocardial infarction; DM: diabetes mellitus; TG: triglyceride; TC: total cholesterol; HDL-C: high-density lipoprotein cholesterol; LDL-C: low-density lipoprotein cholesterol; *LDLR*: low-density lipoprotein receptor; *APOB*: Apolipoprotein B; *PCSK9*: proprotein convertase subtilisin/Kexin type 9; *STAP1*: Signal-transducing adaptor protein 1.

Table S6. Plasma LDL-C levels stratified by genetic mutations in the patients with very early-onset CAD.

**Mutation Type**	**Number of patients**	**LDL-C, mmol/L**
All	105	5.77 ± 3.36
FH mutation-negative	65	4.60 ± 1.62
FH mutation-positive	40	7.65 ± 4.49
LDLR	15	7.46 ± 4.93
APOB	7	4.72 ± 1.05
PCSK9	2	5.71 ± 1.00
STAP1	1	4.67
LDLR Homozygote	4	13.88 ± 4.72
Two mutations	11	8.21 ± 3.23

Data are expressed as mean ± SD, or n (%). LDL-C: low-density lipoprotein cholesterol; CAD: coronary artery disease;*LDLR*: low-density lipoprotein receptor; *APOB*: Apolipoprotein B; *PCSK9*: proprotein convertase subtilisin/Kexin type 9; *STAP1*: Signal-transducing adaptor protein 1.

Table S7. Percentage participants with early-onset CAD meeting clinical and genetic criteria for FH diagnosis based on different initial LDL-C levels.

LDL-C	No.	Simon Broome Criteria (Possible and Definite)	Dutch Lipid Clinic Criteria (Probable and Definite)	Genetic Study
≥ 4.9 (190 mg/dL)	43	18 (41.8%)	26 (60.5%)	27 (62.8%)
≥ 4.6 (178 mg/dL)	49	18 (36.7%)	27 (55.1%)	30 (61.2%)
≥ 4.4 (170 mg/dL)	58	18 (31.0%)	27 (46.6%)	31 (53.4%)
≥ 4.0 (155 mg/dL)	73	18 (24.7%)	28 (38.3%)	37 (50.7%)
≥ 3.8 (145 mg/dL)	77	18 (23.4%)	28 (36.4%)	38 (49.4%)
≥ 3.4 (130mg/dL)	105	18 (17.1%)	28 (26.7%)	40 (38.1%)

Data are expressed as n (%). LDL-C: low-density lipoprotein cholesterol.

Figure S1. Plasma LDL-C levels stratified by genetic mutations in the patients with very early-onset CAD.

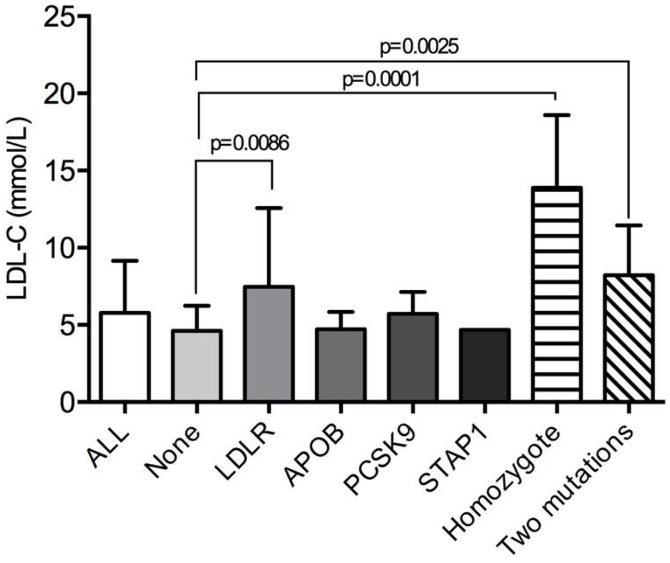

LDL-C: low-density lipoprotein cholesterol; CAD: coronary artery disease. *LDLR*: low-density lipoprotein receptor; *APOB*: Apolipoprotein B; *PCSK9*: proprotein convertase subtilisin/Kexin type 9; *STAP1*: Signal-transducing adaptor protein 1.

Figure S2. Percentage participants with CAD meeting clinical and genetic criteria for FH diagnosis.

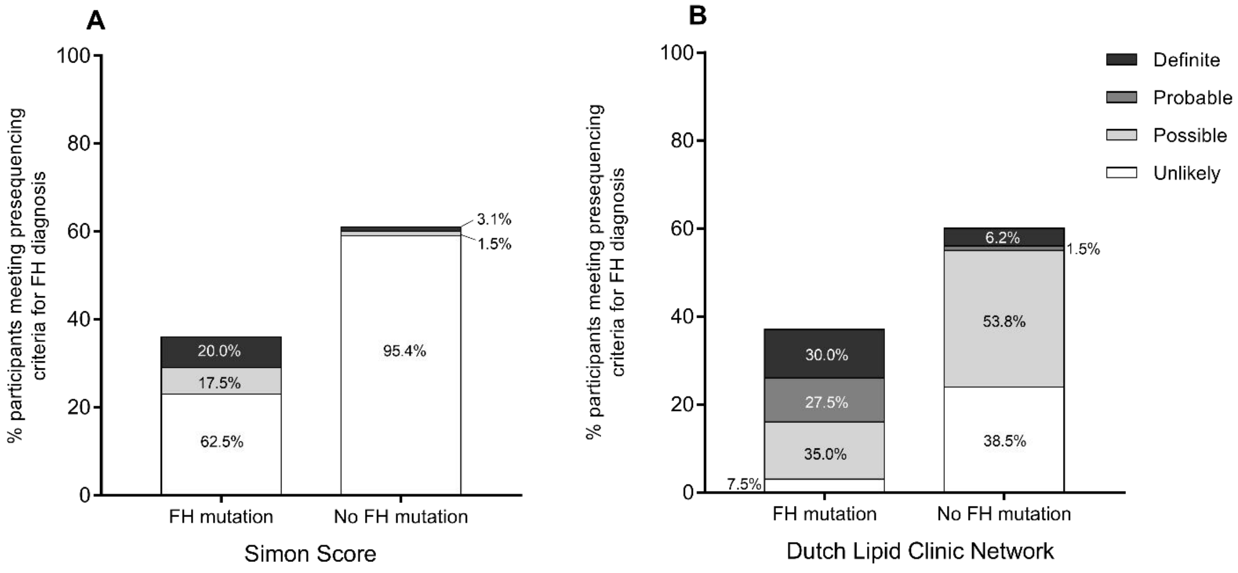

CAD: coronary artery disease; FH: familial hypercholesterolemia.

Figure S3. Receiver operating characteristic curves of LDL-C (n=105).

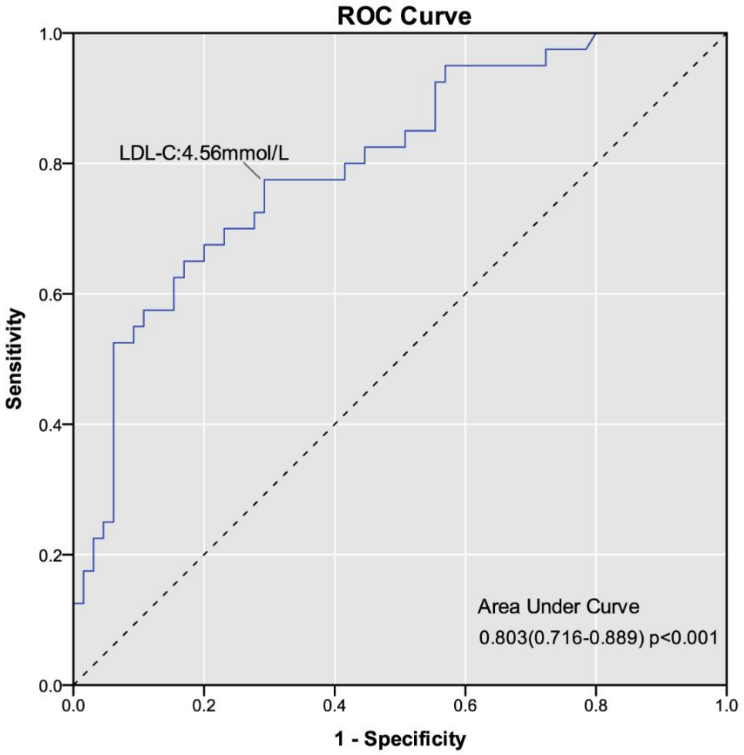

LDL-C: low-density lipoprotein cholesterol. Sensitivity=0.775, Specificity=0.708.
